# Back Pain in Poland and Germany: A Survey of Prevalence and Association with Demographic Characters

**DOI:** 10.1155/2014/901341

**Published:** 2014-07-01

**Authors:** Lea Henn, Katarzyna Schier, Tamara Brian, Jochen Hardt

**Affiliations:** ^1^Medical Psychology and Medical Sociology, Clinic for Psychosomatic Medicine and Psychotherapy, School of Medicine, Johannes Gutenberg University, Duesbergweg 6, 55128 Mainz, Germany; ^2^Faculty of Psychology, University of Warsaw, Ulica Stawki 5/7, 00-183 Warsaw, Poland

## Abstract

*Background.* Back pain is the most common form of pain and leads to high costs in all medical care systems. *Objective.* The present study examines the prevalence of back pain and its associations with some basic demographics. *Methods.* Two samples from Poland and Germany (about *n* = 500 each) were examined via Internet regarding back pain, gender, age, and body mass index (BMI). *Results.* Back pain is more common in women than in men (risk ratio about 1.7), and a high BMI constitutes an additional risk factor. Age was not related to back pain prevalence. *Conclusion.* Congruent results in two countries based on the same measure of back pain lead to the assumption that much of the variety found in estimates of back pain are due to inconsistent assessment. For future research, a definition of common criteria on how to assess back pain would be an asset.

## 1. Introduction

Back pain is the most prevalent pain; estimates say that about 9.4% of the global population are severely affected [1]. It ranks at least since 1990 and till 2010 on the first place for the years lived with disability [2]. Back pain accounts for enormous health care costs: estimates for Germany were about €7000 per year on per affected patient basis or €1322 per person and year based on the population (data from 2004/5: [3, 4]). More than half of these costs are due to absence from work; other costs result from treatment and rehabilitation. Costs due to early retirement were not included in the calculations; however, data from 2012 show that back pain (and other musculoskeletal disorders) is the reason for only approximately 12% of the cases of early retirement nowadays [5]. Even higher costs were reported from the USA, where annual costs resulting from chronic lower back pain patients were estimated at almost $12,000 for medical care alone, with a large proportion coming from in- and outpatient service [6]. The problem of back pain may even increase in future, since members of the western population are showing a decrease in physical activity and an increase in weight over the years (e.g., [7]).

A search in the Cochrane database in January 2014 utilizing the term “chronic back pain” yielded 61 reviews, 19 of those were withdrawn, were protocols, or did not primarily deal with chronic back pain. The remaining 42 reviews cover a wide range of treatments, for example, total disk replacement, radiofrequency denervation, various drugs taken orally or being injected, lumbar support, transcutaneous electric nerve stimulation, traction, physical conditioning, back school, cognitive behavioral therapy, and various more. The essence of the reviews does not provide much hope for back pain patients; most therapies are either not effective in the long term, have side effects, or show little or conflicting evidence to support their effectiveness. Effect sizes generally were small. For clinical practice, this means that the best therapy for each patient needs to be determined individually, and, particularly, for nonspecific back pain, there are not many evidence based recommendations available (e.g., [8]).

Data from the USA indicate that, in practice, treatment mostly includes or even solely relies on pain killers (about 70% of the patients). In the USA weak opioids have been prescribed against back pain more and more since the mid-1990s. In the USA, about 40% of back pain patients sometimes take opioids with strong regional variation [9]. Fortunately, opioid use is less frequent in Europe—only about 25% of chronic back pain patients take them [10]. Other than clinical experience, there is no scientific evidence that opioids work better than other pain killers for moderate back pain [11, 12]. Since 2005, deaths due to prescribed opioids have outnumbered the deaths due to illegal drugs on the street in the USA [13]. It is unclear whether a similar development will occur in Europe. The recommended alternatives here are mostly nonsteroidal anti-inflammatory drugs (NSAID). However, NSAIDs should not be used against back pain for a period longer than three months [8].

Strong reliance on drug therapy for treating back pain is not necessarily optimal. Airaksinen provides a more positive overview than the Cochrane reviews. When back pain becomes chronic, multidisciplinary treatment shows much better results than one-track treatment with pain killers [8]. Multidisciplinary treatment can reach 6-month pre-post effect sizes for pain reduction of about *d* = 0.8; as long as there is no wish for early retirement, the effect is zero or even negative [14]. Also, back pain patients can develop medication overuse headache and in the long term pain killers do not lead to much success in back pain treatment. In the long run, surgical interventions show high rates of complications or nonsuccess. Such considerations have led some professionals to rethink traditional back pain treatment in western medical systems and, for example, suggest incorporating physiotherapists much earlier [15]. A small study comparing 15 patients against controls who trained for ten weeks only two minutes per day showed a promising reduction of back and neck pain [16].

The central aim of the present investigation is to explore the prevalences of back pain in Poland and Germany and their associations with age, gender, and body mass index (BMI).

## 2. Material and Methods

### 2.1. Sample

The analysis was performed on two Internet surveys of 508 subjects in Poland and 500 in Germany. Participants who were registered at a professional marketing company received an email which invited them to take part in the survey (http://www.linequest.de/). The questionnaire set contained about 280 items and participants received compensation of about €4,30 for filling out the questionnaire. The Ethics Commissions of the University of Düsseldorf (2873) and the Landesärztekammer Rheinland-Pfalz approved the project (837.185.07). During data collection, information was displayed at the University of Mainz in German and Poland verifying the scientific background of the study. Demographic data from the samples are displayed in Table 1.

### 2.2. Variables

The central variable “back pain” was assessed via one item of the “symptom check list 27 plus,” a questionnaire designed by Hardt [17, 18]. Back pain is one of 20 symptoms whose occurrence in general was assessed. The answer was a five-point Likert scale containing the categories “never,” “rarely,” “sometimes,” “often,” or “very often.” For the present study, the variable was dichotomized; that is, “never,” “rarely,” and “sometimes” were counted as no back pain and “often” or “very often” as back pain. The rationale behind the dichotomization was to yield a simple response variable, which (a) reflects the subjects suffering mostly under back pain and (b) allows for complex tests of predictors. We did not consider aspects as precise location, that is, upper or lower back pain, pain in limbs associated with the back pain, or duration.

Age, gender, weight, and height were ascertained; BMI was calculated as kg/m^2^. Since the distribution of the BMI had serious outliers to the right, it was coded into four categories, as displayed in Table 1. Two subjects who had missing data on BMI were allotted to the group BMI ≤ 25.

### 2.3. Statistics

A logistic regression analysis was performed on the response variable back pain. Beside the four main effects, all two-way interactions as well as quadratic effects for age and BMI were tested. First, a backward selection of the main effects was performed. Second, the respective interaction and quadratic effect were tested always including all underlying main effects [19]. In case of a quadratic effect, the test had two degrees of freedom (df) in the nominator; while in case of an interaction, there were three df. Significance was set to alpha = 0.01 in order to avoid statistically significant but clinically nonsignificant results. No trends were interpreted. The analysis was performed using Stata [20]. The significant predictors were presented in a graph.

## 3. Results

**Figure 1:**
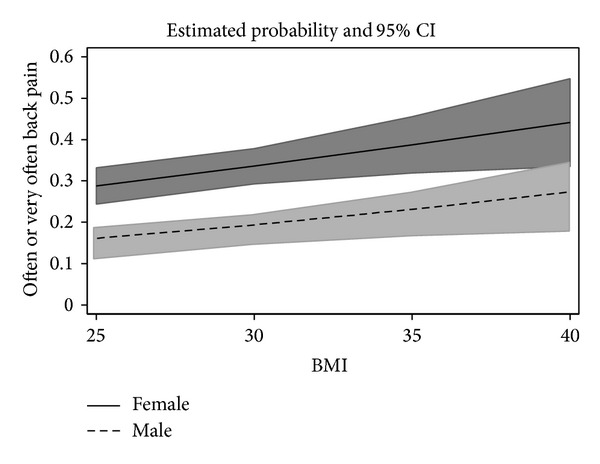
Estimated probabilities (and 95% confidence intervals) for back pain.

In Poland, a total of 22.6% reported to have often or very often back pain, whereas in Germany the corresponding prevalence rate was even 28.8%. Table 1 shows the comparisons of the samples from Poland and Germany in detail. Polish participants were about six years younger on average than the German ones, had a higher BMI, and were more often married and a higher professional status was reported. Other differences were small and did not reach significance.  Table 2 shows the result of the logistic regression analysis. Two predictors for back pain could be identified: gender and BMI. The gender effect was strong and explained about 2% of the variance in the logistic regression. Women were more likely to report back pain than men. The effect for BMI was relatively small and explained only 0.75% of variance. However, the direction is plausible: the higher the BMI, the higher the likelihood for reported back pain. There was no interaction between gender and BMI; the *P* value was 0.237. Accordingly, Figure 1 displays the two main effects, not including the interaction effect. No other effects reached significance. The closest value would have been the main effect for country (*P* = 0.029).

## 4. Discussion

The prevalence of back pain is higher in our estimate than in others. For example, Hoy et al. [1] estimated a prevalence of about 9.4%. The comparability of prevalence estimates suffers because of the various definitions of pain that exist [21]. For example, 12-month estimates for back pain were 66% for women and 58% for men [22]. Such high prevalence estimates usually include subjects with mild or moderate pain and/or relatively rare pain. So, for the present estimate, one should be aware that some participants with mild or moderate pain are included.

A central result is that women have back pain more often than men. In the low BMI group (≤25), the point estimates were 29% and 16%, so the back pain prevalence ratio between women and men is 1.8. In the highest group (BMI 40 plus), the ratio was lower at 1.6, but there is still only a minimal overlap in the confidence intervals between women and men (Figure 1). Such strong gender differences in back pain have been reported in other studies (e.g., [23]), but the average gender difference for back pain in epidemiologic studies is smaller [24]. It may be worthwhile to examine for which factors pronounced gender differences exist and for which ones they do not.

A further interesting result is the absence of any age effect in the present study. This also stands in some contrast to most other studies (e.g., [24]), where generally an increase of the back pain prevalence is seen from individuals in their 20s compared to those in their 60s, followed by a plateau or even decrease. Kędra and Czaprowski describe interesting data that may explain this phenomenon [25]. In youths (age 10–19) they observed no age trend when asking about back pain frequency, but a strong increase in back pain severity was observed. As possibly with gender, the various characteristics may show different associations with age.

An association of back pain with BMI is obvious, since a heavy build strains the spine. It was smaller than expected and we would have assumed an interaction of BMI with age additionally. The rationale behind this was that carrying heavy weight over a long period of time should be more destructive for the spine than a shorter time could be. This was not the case here; the *P* value of the interaction effect was far too large to be of interest.

There was no effect for country in the present survey indicating that the same question leads to the same result in different countries, at least regarding Poland and Germany. This was shown not only for the prevalence estimate (where the difference almost reached significance), but also for the associations with gender, age, and BMI.

The present study has the following limitations. (1) Data rely on self-reports and are not verified by medical experts. (2) Data were collected via Internet surveys. It is not known how far they are representative for the population. However, there was a bias towards higher education. (3) We focus on three predictors, here, that is, age, gender, and country. Many more factors probably contribute to back pain, for example, coping [26] or behavioral factors or stress (e.g., [27]). (4) The results of this cross-sectional study do not reflect any causality. Both associations with back pain, the one for BMI and the one for gender, are likely to be mediated by various factors, for example, lack of physical exercise, diet, or genetics.

## 5. Conclusion

The present study shows congruent back pain estimates in Poland and Germany, not only regarding prevalence but also concerning associations with gender, age, and BMI. The results are partly congruent to others reported in the literature and differ in some respects. Those results underline the necessity of a common classification for back pain and preferably for other forms of pain as well (e.g., [28]).

## Figures and Tables

**Table 1:** Demographics.

Variable	*N* = 508	*N* = 500		*P*
Country	Poland	Germany		
Sex (% female)	56.30	50.00	*χ* ^2^_(1)_ = 4.02	0.045
Age: $\overset{-}{x}$ (sd)	38.7 (14.4)	44.8 (17.1)	*t* = 6.61	<0.001
Body mass index (%)				
≤25.00	55.9	44.4	*χ* ^2^_(3)_ = 27.75	<0.001
25.01–30.00	32.5	35.0		
25.01–30.00	7.7	15.8		
>30.00	3.9	4.8		
Back pain (%)	22.6	28.8	*χ* ^2^_(1)_ = 5.01	0.025
Current partner in life (%)				
Married	48.8	43.0	*χ* ^2^_(4)_ = 16.71	0.002
Stable relationship >6 months	23.8	23.2		
Stable relationship ≤6 months	3.5	3.8		
No stable relationship	17.3	26.6		
Other	6.5	3.4		
Profession (%)				
(I) Higher-grade professional, administrator, or manager	14.6	4.2	*χ* ^2^_(6)_ = 80.12	<0.001
(II) Lower-grade professional, administrator, or manager; higher-grade technician	40.2	30.2		
(IIIa) Skilled nonmanual employee	18.1	33.0		
(IIIb) Skilled manual employee	9.2	5.2		
(IV) Partly skilled worker	4.7	10.8		
(V) Unskilled labourer	6.7	6.2		
Others: housewife, housemen	6.5	10.4		

**Table 2:** Regression analyses for “often or very often back pain.”

Explanatory variable	Odds ratio	Standard error	*z*-value	*P* value	Δ*R* ^2^
Variables in the equation, pseudo *R* ^2^ = 2.55%					
Constant	0.11	0.06	−4.48	<0.001	
BMI	1.05	0.02	2.95	0.003	0.75%
Gender	0.48	0.07	−4.81	<0.001	2.09%

Terms not in the equation, value if added next					
Country	1.38	0.21	2.18	0.029	0.42%
Age	1.00	0.005	0.58	0.559	0.03%
Age^2^	1.00	0.0003	−0.69	0.490	0.07%
BMI^2^	1.00	0.004	−0.16	0.874	0.00%
Country ∗ gender	0.94	0.27	−0.20	0.843	0.42%
Country ∗ age	1.00	0.01	0.05	0.963	0.42%
Country ∗ BMI	0.98	0.03	−0.53	0.597	0.44%
Gender ∗ age	1.00	0.01	−0.07	0.944	0.03%
Gender ∗ BMI	0.96	0.04	−1.18	0.237	0.12%
Age ∗ BMI	1.00	0.001	0.77	0.433	0.08%
